# Decreasing the physical gap in the neural-electrode interface and related concepts to improve cochlear implant performance

**DOI:** 10.3389/fnins.2024.1425226

**Published:** 2024-07-24

**Authors:** Joseph T. Vecchi, Alexander D. Claussen, Marlan R. Hansen

**Affiliations:** ^1^Department of Molecular Physiology and Biophysics, Carver College of Medicine, Iowa City, IA, United States; ^2^Department of Otolaryngology Head-Neck Surgery, Carver College of Medicine, Iowa City, IA, United States

**Keywords:** cochlear implant, neural electrode, regenerative medicine, hearing loss, biomaterials, optogenetics, neural prostheses, spiral ganglion neuron (SGN)

## Abstract

Cochlear implants (CI) represent incredible devices that restore hearing perception for those with moderate to profound sensorineural hearing loss. However, the ability of a CI to restore complex auditory function is limited by the number of perceptually independent spectral channels provided. A major contributor to this limitation is the physical gap between the CI electrodes and the target spiral ganglion neurons (SGNs). In order for CI electrodes to stimulate SGNs more precisely, and thus better approximate natural hearing, new methodologies need to be developed to decrease this gap, (i.e., transitioning CIs from a far-field to near-field device). In this review, strategies aimed at improving the neural-electrode interface are discussed in terms of the magnitude of impact they could have and the work needed to implement them. Ongoing research suggests current clinical efforts to limit the CI-related immune response holds great potential for improving device performance. This could eradicate the dense, fibrous capsule surrounding the electrode and enhance preservation of natural cochlear architecture, including SGNs. In the long term, however, optimized future devices will likely need to induce and guide the outgrowth of the peripheral process of SGNs to be in closer proximity to the CI electrode in order to better approximate natural hearing. This research is in its infancy; it remains to be seen which strategies (surface patterning, small molecule release, hydrogel coating, etc.) will be enable this approach. Additionally, these efforts aimed at optimizing CI function will likely translate to other neural prostheses, which face similar issues.

## 1 Introduction

Neural prostheses replace or enhance neural pathways that are diminished or absent due to disease, trauma, or aging. These devices have improved greatly in recent years; however, they fail to fully emulate the native neural pathways they seek to restore (Soleymani et al., 2016; Jiam et al., 2017). Enhancing the neural-electrode interface is an essential element of next generation of neural prostheses (Senn et al., 2017; Li C Y, et al., 2021). Poor tissue integration of the electrode array limits the function of these implants; to improve outcomes, future devices will need to recapitulate the architecture and function of native neural systems more accurately.

**Figure 1:**
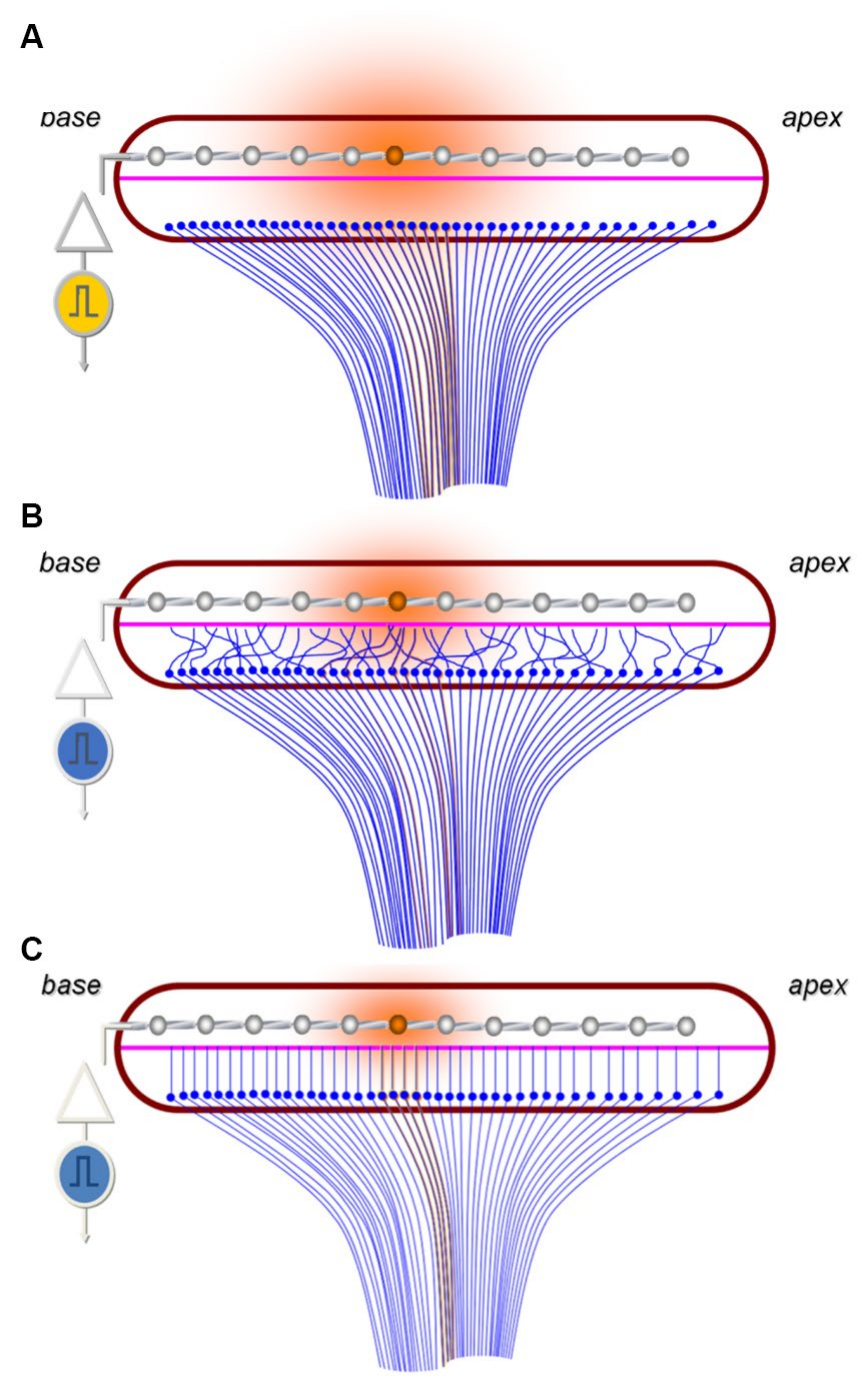
Schematic highlighting the challenges of tonotopic specificity of CIs. **(A)** Image representing current paradigm of how CI electrodes (silver) stimulate (orange) the SGNs (blue) across the tonotopic axis of the cochlea in a region of devoid of functioning hair cells. Note the large distance between the electrodes and the SGNs resulting in broad current spread and SGN stimulation (shaded orange circle). **(B)** Hypothesized outcome of non-guided SGN outgrowth toward the electrodes which results in random overlap and stimulation of SGNs encoding a variety of frequencies. **(C)** Image representing idealized guided SGN neurite outgrowth into close proximity the electrode array which enables more specific SGN stimulation and reduced electrical current demands.

An example of a highly successful neural prosthetic is the cochlear implant (CI). A CI replaces the mechanosensory transduction of sound within the cochlea by directly stimulating spiral ganglion neurons (SGNs) to provide auditory sensation. CIs are revolutionary in that they restore hearing for those with moderate to profound sensorineural hearing loss, however, they are limited by the multiple-fold difference in independent perceivable frequency channels they provide relative to the normal cochlea (Berg et al., 2019). Many drivers contribute to the observed shortcomings in CI performance, including limitations in cortical plasticity, dynamic range representation, and structural changes to the auditory pathway in those receiving CI (Limb and Roy, 2014; Pisoni et al., 2017). A major factor contributing to the limitation in performance is the large physical distance between the electrode surface and the target SGNs (Figure 1A; Shi et al., 2021; Ertas et al., 2022). For reference, the CI electrode array is inserted into the scala tympani and typically positioned hundreds of microns away from the target SGNs in the modiolus, compared to the tens of nanometers of the native inner hair cell(IHC)-SGN synapse (Wang et al., 2015; Moverman et al., 2023). Due in part, to the large gap between the electrode and target neurons, current spreads significantly over this distance. This results in overlap of activation of SGNs across adjacent electrodes and thus, a limitation in the number of discrete, useable channels by the user. Overall, this physical gap represents a major limitation of CI performance, leaving current CI performance plateaued with 8 or 16 independently distinguishable channels, a resolution much lower than native hearing (Boisvert et al., 2020). Given this, there is intense motivation to develop methodologies that result in the electrode array being able to stimulate the nervous system more precisely.

The large physical distance between the electrode and the neurons is not the only anatomical limitation of CIs. First, upon implantation the electrode array becomes engulfed in a fibrous capsule with neo-ossification as a result of inflammatory response to biomaterials (Rahman et al., 2022). This capsule increases electrode impedance and thus may also fundamentally limit CI performance (Wilk et al., 2016; Briggs et al., 2020). Additionally, it may be particularly challenging to selectively stimulate low frequency encoding SGNs in the apex with a CI. This is because the apical bulb of the spiral ganglia is condensed and particularly crowded with neurons where four octave bands of frequency are perceived within 3.4 mm of spiral ganglia (Pamulova et al., 2006; Li H, et al., 2021). These same four octave bands are sensed over approximately 4 times the length of the basilar membrane (Li H, et al., 2021). Thus, a CI electrode that stimulates these neurons from a perimodiolar position will be unable to precisely target this region of the cochlea to allow perceptual differentiation of low frequency sounds. Benefits of preserving the frequency specificity in the apical region are evidenced by improved outcomes in CI patients with preserved residual hearing. CI patients with preserved apical hearing (and thus, frequency specificity) show improved auditory performance, particularly in challenging listening situations such as speech understanding in noise and music appreciation (Büchner et al., 2009; Gantz et al., 2018, 2022; Shim et al., 2023). Likewise, beyond retaining apical acoustic function, preservation of the peripheral axons of apical SGNs that project into the basilar membrane will theoretically enable better spatial resolution assuming these elements of the SGNs are functional and responsive to the electrical stimulation.

Given these challenges, there is interest to study and implement new methodologies which result in the electrode array being able to stimulate the nervous system more precisely. These techniques broadly range from modifying the electrode array shape, altering the surgical placement, inhibiting the immune/inflammatory response, and leveraging regenerative medicine, among many others. An intriguing approach seeks to induce the outgrowth of neurites toward the basilar membrane, thereby mimicking the native distribution of peripheral SGN axons across the apex of the cochlea. This approach would transition CIs from a far-field to near-field device and improve CI resolution. Therefore, work into how to induce the SGN neurites to grow into close proximity to the CI electrode and to improve the neural—electrode interface in CI, especially in the cochlear apex, has been proposed. This review will focus on describing the efforts to guide SGN neurite growth into closer proximity to the CI electrode as well as a brief discussion of other approaches in CI and auditory regeneration also involving engineering the neural-electrode interface.

## 2 Guiding SGN neurite growth into close proximity to the CI electrode

The requirements for guiding SGN neurites to grow into close proximity to the CI electrode can be divided into 4 broad components. These include: (1) initiating regenerative neurite outgrowth, (2) precisely guiding this *de novo* neurite growth, (3) stopping the outgrowth near or at the electrode interface, and (4) myelinating the new neurites. In the discussion of these below, the state of the current research and how they would translate to CI are examined.

### 2.1 Initiating regenerative neurite growth

The peripheral axonic processes of type 1 SGNs extend into the organ of Corti along the basilar membrane and are the site of initial electrical activity induced by synaptic vesicle release from inner hair cells (IHCs) in response to sound. SGN somata are positioned in the spiral ganglion within the modiolus. The peripheral processes of SGNs in cochleae receiving CIs may not be synapsed with IHCs and may be retracted, due to disease or damage; regardless of the current position of the peripheral axon, *de novo* neurite outgrowth will need to be initiated to grow processes toward the electrodes. SGNs have minimal ability initiate spontaneous outgrowth, especially in adult mammalian systems (Schwieger et al., 2015; Zhang et al., 2022). Therefore, it is expected that factors to induce neurite growth are needed for this application.

#### 2.1.1 Growth factors

A variety of factors induce the outgrowth of SGN neurites. The most widely used approach provides exogenous neurotrophins capable of activating tropomyosin receptor kinase (Trk) receptors. Type 1 SGNs express TrkB and TrkC receptors, which are typically activated by brain derived neurotrophic factor (BDNF) and neurotrophin-3 (NT-3), respectively. While there are specific functions attributable to each factor (Schimmang et al., 2003; Flores-Otero et al., 2007; Green et al., 2012; Szobota et al., 2019), they show overlap in function and ability to promote SGN outgrowth and synaptogenesis. Nevertheless, the differences in expression and function are important to consider. For example, they have different tonotopic expression patterns, with TrkB/BDNF more prominent than TrkC/NT-3 in the base and the inverse for the apex. These differences likely contribute, at least in part, to specific tonotopic firing patterns of SGNs (Davis, 2003; Flores-Otero and Davis, 2011). Additionally, BDNF better stimulates neurite outgrowth, while NT-3 better promotes innervation and synaptogenesis (Green et al., 2012). Thus, for the purpose of initiating growth, BDNF is a common choice. Importantly, the effects on exogenous neurotrophic factors on SGN firing patterns across the tonotopic axis, particularly those induced by electrical stimulation should be considered. Other growth factors also stimulate regenerative neurite outgrowth in SGNs including, but not limited to, ciliary neurotrophic factor (Schwieger et al., 2015), fibroblast growth factor 8 (García-Hernández et al., 2013), somatotropin (Gabrielpillai et al., 2018), and glial cell-derived neurotrophic factor (Euteneuer et al., 2013). These factors are small proteins and present challenges for long-term delivery to the cochlea. Further, controlled release of these factors in the setting of a CI will likely require shelf stability and/or solvents to encapsulate the compound in order have necessary control of release (Hendricks et al., 2008; Liu and Yang, 2022). To bypass these hurdles, genetically engineered cells that produce neurotrophic factors could be co-implanted with the electrode or the CI electrode used to electroporate exogenous genes encoding neurotrophic factors into native cochlear tissue which would, in turn, produce the factor (Pinyon et al., 2014).

#### 2.1.2 Chemicals

To overcome the challenges inherent to sustained delivery of functional peptides or proteins, chemical analogs that target receptors are also being explored. Currently, Trk receptor agonists appear to be the most popular and promising venture. Indeed, small molecules capable of activating (i.e., phosphorylating) TrkB promote SGN outgrowth *in vitro* and SGN survival and function *in vivo* (Yu et al., 2012, 2013). Similar work has also shown promise in targeting TrkC (Kempfle et al., 2021). Further effort is needed to validate these compounds to ensure that they adequately and specifically target the receptor (Pankiewicz et al., 2021) and to explore the methodologies to control the release in the cochlea (Sun et al., 2022; Wulf et al., 2022). Beyond Trk agonists, other small molecules are also being explored. These include molecules that activate pathways known to promote neurite outgrowth and pharmacological screens to repurpose already approved drugs (Whitlon et al., 2015).

### 2.2 Guiding *de novo* neurite growth

**Figure 2:**
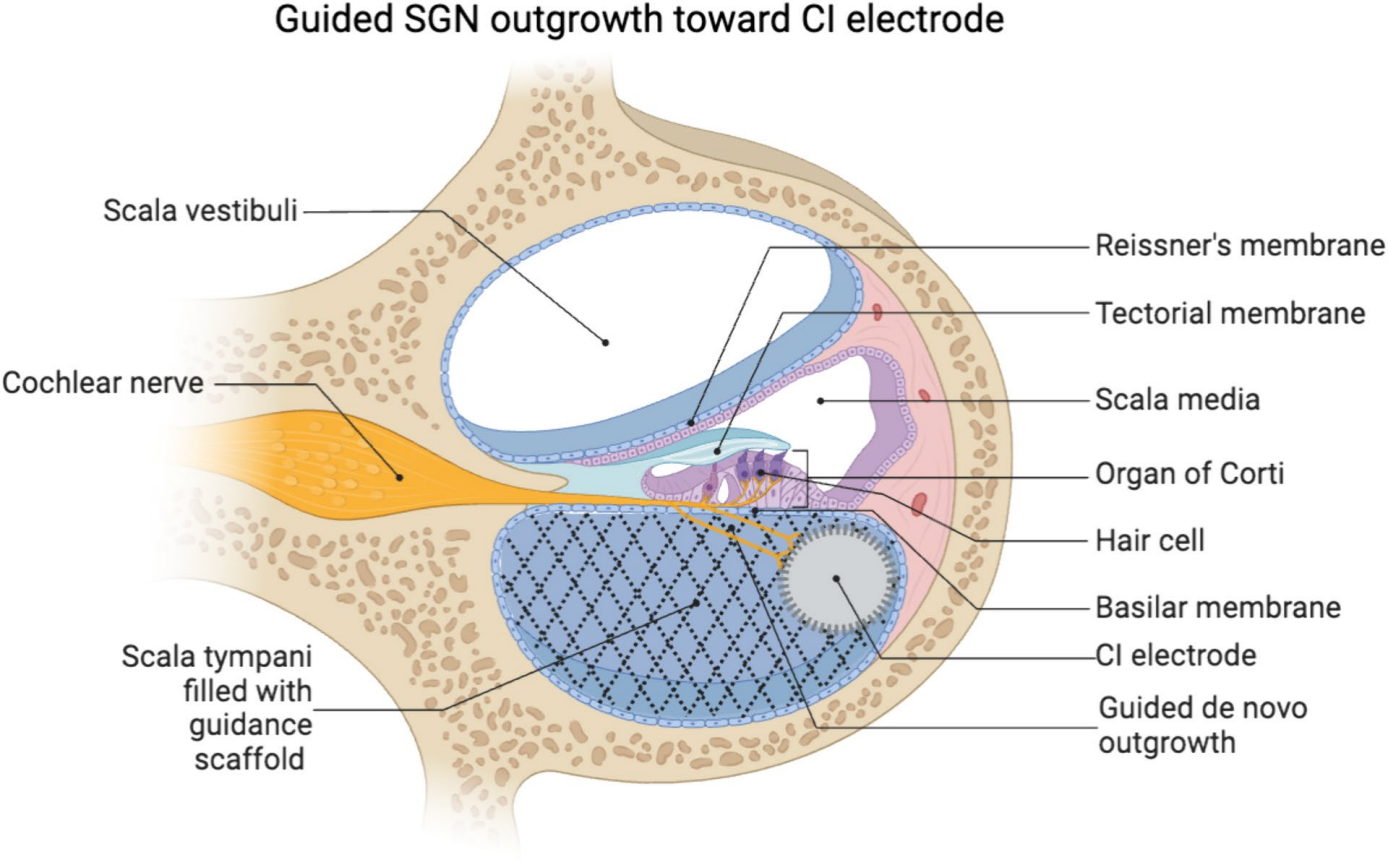
Schematic of a scaffold in the scala tympani guiding *de novo* neurite growth toward a lateral wall positioned CI electrode array. Created using biorender.

Once neurite outgrowth is initiated, there are two essential considerations: (1) the neurites must be guided to maintain the precise tonotopic organization of the native cochlea innervation, and (2) to reach the CI electrode array in the scala the neurites must exit the organ of Corti and traverse into the scala tympani, which is normally filled with perilymph and devoid of cells or extracellular matrix. In combination, both of these factors necessitate the use of organized guidance cues to guide this *de novo* neurite growth (Figure 2).

To appreciate the need for guidance cues, it is helpful to understand how the tonotopic architecture of the cochlea enables natural hearing and why this layout will likely need to be maintained for effective neural regeneration. Sound energy induces a traveling wave in the basilar membrane in a tonotopic pattern based on frequency, with high frequencies stimulating the base and low frequencies the apex of the cochlea. The IHCs stimulated by specific frequencies translate this mechanical signal of sound into an electrical nervous signal via release of synaptic vesicles to target SGNs specific for those IHCs. The SGNs then relay this signal specific to a given frequency into the cochlear nucleus in the brain. This precise layout enables the perception of continuous sound frequencies over the approximately 3,500 IHCs.

Given the importance of the tonotopic architecture in function of the auditory system, it is expected that this organization will need to be preserved during regenerative approaches. For example, in the context of neurite outgrowth toward a CI electrode array, as the peripheral processes of the SGNs extend into close proximity to the CI electrode they should maintain their position relative to one another to preserve the tonotopic patterning underlying frequency perception. If the outgrowth becomes spatially disorganized or random with processes crossing over each other, then the precise tonotopic specificity will likely be lost (Figure 1B). If this architecture is compromised, neurons corresponding to a variety of frequencies would be stimulated by specific electrodes, thus diminishing signal resolution. It is important to note that this concept is theoretical and implied by related work (Guiraud et al., 2007; Walia et al., 2023), though it remains to be seen if future CI could be programmed to overcome disorganized outgrowth via intelligently programming each electrode to the frequency of the neurons that it stimulates (Grasmeder et al., 2014).

Bioengineering techniques will likely be required to provide guidance cues such that as outgrowth is stimulated neurite overlap is minimized and tonotopic architecture is preserved (Figure 1C). Many strategies have been proposed and evaluated, including diffusible chemical gradients (Coate et al., 2015; Li et al., 2017), patterned peptide surface coatings (Tuft et al., 2018; Truong et al., 2021), and engineered surface topography (Tuft et al., 2013; Hu et al., 2022), among others (Druckenbrod et al., 2020). Of these, the most commonly reported *in vivo* technique is to use chemical gradients of neurotrophic factors (Budenz et al., 2012; Sameer Mallick et al., 2013). This approach involves a coating on the electrode intended to elute the factor (Kikkawa et al., 2014), or the implant may be co-implanted with cells (Rejali et al., 2007) or particles (Gunewardene et al., 2022) that deliver the factor. When used *in vivo*, chemical gradients of these factors have been shown to not only be neural protective, but also to induce *de novo* outgrowth from the peripheral process of SGNs toward the CI electrode when neurotrophic factors are co-delivered (Wise et al., 2011; Landry et al., 2013). However, it is not clear whether diffusible gradients alone will suffice to provide precise neurite guidance.

As an alternative to or combinatory approach with chemical gradients, engineered surface patterning can effectively direct cell material interactions and guide neurite growth as well, often with much greater precision (Tuft et al., 2018). There is tremendous diversity of types of patterns (chemical, protein, biophysical, topographical) and approaches to make these patterns. First, engineers have devised methods to create biochemically patterned surfaces. Methods to do this include photo-deactivation (Leigh et al., 2017b), protein immobilization (Sorribas et al., 2002; Kim et al., 2019), microcontact printing (Eichinger et al., 2012; Joo et al., 2015), and creation of patterned zwitterionic coatings (Leigh et al., 2017a). Each of these methods restricts protein absorption to selective regions of the substrate. By using precise spatial patterning of zwitterions, chemo-permissive proteins, and/or chemo-repulsive proteins, it is possible to spatially direct cell adhesion, neurite guidance, and neurite repulsion (Humenik et al., 2021; Truong et al., 2021). Importantly, further work is needed to translate these 2D *in vitro* approaches to *in vivo* application.

Separately or in combination with the biochemical approaches (Truong et al., 2021), biophysically patterned substrates can be generated in a variety of approaches. These include electrospinning, lithography (Tonazzini et al., 2020), or selective photopolymerization via photomasks or two-photon polymerization (Marino et al., 2013; Tuft et al., 2013). Biophysical patterning offers a particular advantage in the inherent stability of the polymer systems. The polymers used to make these patterned surfaces are shelf stable and are already used in a variety of biomedical implants. An additional benefit is that the feature geometry for these substrates can be controlled on the submicron scale (Tuft et al., 2013; Limberg et al., 2022). Thus, these systems enable tremendous control of cell behavior with precise modifications and reproducibility in creating consistent materials (Vecchi et al., 2024). Another potential benefit of engineered biomaterials is that they provide a substrate for *de novo* SGN outgrowth through the perilymph. Traditional CI electrodes are positioned in the scala tympani, thus for the peripheral projections of SGNs to grow into close proximity to the electrode the neurite would likely need to extend from the organ of Corti or osseus spiral lamina and, possibly through the perilymph. The perilymph is liquid and the normal scala tympani is an environment not suitable for cell survival. Alternatively, the fibrotic capsule that encapsulates the electrode array (Foggia et al., 2019; Rahman et al., 2022) could be engineered to provide a scaffold that supports and guides *de novo* neurite growth. Therefore, it has been suggested to use polymeric biomaterial systems to provide a suitable substrate to support SGN neurite outgrowth (Senn et al., 2017; Li C Y, et al., 2021; Figure 2). The principles learned from the patterned 2D systems could inform the design of a porous 3D coating of the electrode to induce and guide SGN neurite growth. Future engineered materials may employ the controlled release of neurotrophic factors, curated pore size and stiffness, and incorporate tailored ECM proteins and/or glial cells (e.g., Schwann cells) in order to induce, channel, and permit neurite growth toward the electrode, respectively. Importantly, beyond the effects of an inflammatory/foreign body response to biomaterials, filling the scala tympani with noncompressible material will impede the motion of the traveling wave of the basilar membrane and exacerbate loss of acoustic hearing (Choi and Oghalai, 2005). Therefore, consideration and greater work on the effect of introducing biomaterials to the scala is needed, especially in the context of those seeking to maximize preservation of residual hearing.

Despite the potential of the approaches described above, reports of clinical translation of 3D patterned coatings on CI electrodes to guide neurite growth are limited. Although such a feat has been shown to be possible *in vivo* (Senn et al., 2017), there have not been large scale studies that assess complex outcomes. There are countless *in vitro* pursuits underway (Nella et al., 2022; Wille et al., 2022), as well as many studies aimed at directing neurite growth *in vivo* for other fields of neuro-regeneration (Dixon et al., 2018; Jiang et al., 2020; Zhang et al., 2020). However, the unique challenges of the cochlear environment and limitations of the SGNs may necessitate development of other techniques if inducing and controlling SGN neurite outgrowth proves to be too challenging. One strategy to bypass this hurdle is to co-introduce stem cells, Schwann cells, or other cells to the scala tympani with the electrode array to act as a bridge between the electrode and SGNs (Nella et al., 2022). Theoretically, in this approach, the cells would be co-implanted with the CI. Further, in the case of stem cells or glial cells, these could even be engineered to differentiate into neural cells that synapse with the SGNs with one projection while the other projection would remain in close proximity to the electrode. Similarly, others have explored implanting “living electrodes” containing neurons and axon tracts for cortical implants wherein the implanted neurons extend their projections into the nervous tissue to better integrate the implant (Adewole et al., 2021). While complex, this schema may be advantageous since the stem cells are more dynamic and able to controlled with more precision than SGNs.

### 2.3 Stopping the outgrowth at the CI interface

Another consideration is that the stimulated outgrowth may need to be stopped at, or very near to, the target electrode(s). Similar to the previous discussion on tonotopy, engineering this aspect may be just as important for maintaining tonotopy as guiding the outgrowth. Even if the *de novo* outgrowth of the peripheral processes of the SGNs is perfectly guided, continued growth beyond the electrode interface would likely result in a loss of tonotopy (Figure 1B). Therefore, it is expected that the peripheral projection may need a proper “stop” signal at or near the electrode interface. Importantly, it remains unclear how essential this may be as some studies suggest this aberrant growth may not have an effect if not too exuberant (Landry et al., 2013). Additionally, it is unknown whether efforts at neurite outgrowth would durably persist from the mechanical forces of CI revision surgery, including CI explanation and reimplantation.

While there is not much specific work in the field of CI related to strategies to halt neurite growth near the target, other applications of neural electrodes have investigated such strategies. In particular, prior work has focused on the use of tracts to guide outgrowth, and the controlled application of electrical or optical signal has been used to direct the formation of an artificial synapse at the end of the tract (Bieberich and Guiseppi-Elie, 2004; Shi et al., 2016). In deep brain stimulation approaches, where there may not be as clearly defined a tonotopic architecture to mimic, several approaches seeking to maximize neural contact are being explored (Serruya et al., 2018; Adewole et al., 2021). Overall, depending on the successes of these engineering focused approaches, the field may need to seek to recapitulate how the peripheral process of the SGN naturally forms a synapse with IHCs. The signals resulting in the ribbon synapse may need to be incorporated in the electrode surface for optimal integration of the neural-electrode interface (Coate et al., 2019).

### 2.4 Myelinating new neurites

A last consideration for the complex task of guiding neurite growth is that the native peripheral processes of SGNs are myelinated. This myelination is critical for the health and function of the SGNs, as demonstrated by the fact that auditory circuit myelination happens at the same time in development as when the auditory system functionally develops. Additionally, axonal myelination enables the rapid conduction and temporal precision of electrochemical signals necessary for coding of SGNs and auditory function (Long et al., 2018). Thus, if *de novo* outgrowth is initiated and guided, these extended neurites may need to be myelinated to mimic the native architecture, function, and neural health, as prior research has indicated auditory nerve demyelination disrupts auditory sensation (Wan and Corfas, 2017; Resnick et al., 2018). Importantly, in addition to having the ability to regenerate, the peripheral nervous system also has the ability to remyelinate peripheral sensory neurons (Zhou and Notterpek, 2016). Thus, promotion of re-myelination remains a focus for many in the field of regenerative medicine (Taveggia et al., 2010).

It is not clear how existing (re)myelination approaches will transfer to the cochlea or if these strategies are necessary. However, if remyelination is necessary, Schwann cells could be included with the implanted device and would need to extend with the neurites or cells, and subsequently myelinate the neurons. As a more likely alternative, Schwann cells may lead the way for the regenerative outgrowth of the neurons. That is because these cells are (1) more dynamic than the SGNs, (2) they similarly follow guidance cues, and (3) the presence and orientation of Schwann cells promotes both neurite growth and alignment (Thompson and Buettner, 2006; Bruder et al., 2007; Clarke et al., 2011; Jeon et al., 2011; Tuft et al., 2013). Further a reciprocal signaling network exists between cochlear Schwann cells and SGNs (Hansen et al., 2001). Thus, a reasonable approach to guide neurite growth through the perilymph could be to leverage the regenerative capacity of Schwann cells, wherein Schwann cells support SGN neurite growth, align precisely to biopatterns, and myelinate the regenerated processes (Whitlon et al., 2009; Clarke et al., 2011; Jeon et al., 2011). Overall, it is important that future *in vivo* studies on CI performance with regenerated SGN neurites incorporate the assessment of myelination and the behavior or Schwann cells, as this could significantly impact device function.

## 3 Other relevant approaches to improve CI neural-electrode interface

The stepwise description above does not cover the entire body of research aimed at enhancing CI through improving the neural-electrode interface. Numerous other approaches exist for improving CI as well as ideas to bypass or supplement the immense challenge described in the previous section. These endeavors include: exploring alternative methods for stimulating the SGNs, mitigating the foreign body response to the electrode implanted in the cochlea, and employing genetic or regenerative medicine approaches.

### 3.1 Optical stimulation

A limitation of CI is the reliance on electrical stimulation to transmit signals to the SGNs. Electrical signals exhibit significant current spread *in vivo* (Zeng, 2004), limiting the precision by which CI electrodes can target SGNs. Furthermore, electrical stimulation provided by traditional CI electrodes has a narrower dynamic range compared to natural hearing (Zeng et al., 2002). As a result, CI users often face challenges in distinguishing soft from loud sounds. These two factors not only constrain the actual functionality of CI but also set a theoretical ceiling on their performance. Consequently, researchers are exploring alternative methods of stimulation.

In particular, optical signals, or light, offer advantages over electricity in the aspects mentioned above (Dieter et al., 2020). Optogenetics presents a potential alternative to traditional electrical CI. Optogenetics involves transfecting genes that encode light-sensitive channels, known as opsins, into neurons, and then stimulating the neurons in a controlled manner with a specific wavelength of light (Emiliani et al., 2022). In the context of CI, this approach would entail transfecting SGNs with genes encoding opsins and using an array that emits optical signals (optode) instead of electrical stimulation (Moser and Dieter, 2020). While this approach shows promise, it requires further optimization in key areas.

First, optical stimulation addresses one of the weaknesses of electrical stimulation mentioned earlier, as optodes exhibit much greater spectral selectivity than traditional electrodes (Dieter et al., 2020; Keppeler et al., 2020). Regarding other measures of CI performance, optodes seem to perform as well as electrical electrode in CI in terms of dynamic range but exhibit lower temporal precision (Dieter et al., 2020). In particular, the firing rate of opsins do not approach that needed for natural sound, however, newer engineered opsins partially overcome this rate limitation (Keppeler et al., 2018; Sridharan et al., 2022; Mittring et al., 2023).

An additional significant challenge in optogenetic approaches is the technical difficulty of transfecting the opsin into the SGNs. It remains uncertain what the transfection efficiency would be in adult patients, especially since permanent opsin expression would be necessary. It is also not known what effect the well-documented post-implantation fibrosis and neo-ossification might have on light transmission necessary for optogenetic stimulation. Future research in this field should investigate transfection efficiency in adults and explore engineering improvements in the opsins used to enhance dynamic range and temporal precision in sound encoding (Huet et al., 2021).

A second optical based approach is to use infrared light delivered by a pulsed laser to stimulate firing of SGNs. Neural activation results from spatially and temporally heat induced stimulation of action potentials (Xu et al., 2021). As with optogenetics, infrared stimulation provides significantly enhanced spatial resolution and is used clinically to modulate cerebral cortex activity. The advantage of infrared stimulation over an optogenetic approach is that it does not require exogenous gene transfer, as neural tissue is naturally sensitive to infrared light pulses. Nevertheless, there are still significant hurdles to adopt this technology into a cochlear prosthetic (Xu et al., 2021). Importantly, it could be feasible to combine light emitting optode arrays with electrode arrays to leverage the advantages of each. However, these optical approaches face some of the same challenges of inflammatory tissue responses incurred with electrode arrays.

### 3.2 Mitigating the inflammatory response

A separate critical challenge that needs to be addressed in all implanted biomaterials including electrode and optode arrays is the inflammatory response. Specifically, an inflammatory/foreign body response encapsulates implanted electrodes with a dense fibrous capsule and, in some cases, bone, resulting in several adverse effects on CI device function. There are many negative effects of this foreign body response include wide reaching consequences from: requirement for increased electrical current, loss of residual hearing after implantation, and device failure (Foggia et al., 2019; Simoni et al., 2020). Importantly, the causes of fibrosis in CI are multifaceted, including the immune system’s response to three interrelated components of CI: (1) surgical trauma, (2) implanted materials, and (3) electrical stimulation. Furthermore, regardless of the cause of the inflammatory response, the regenerative approaches discussed thus far will not be possible in the presence of dense fibrous capsule and compromised anatomy secondary to inflammation. In this section, the approaches to mitigate the inflammation resulting from each of these factors are described.

First, the practice of “soft surgical” techniques for atraumatic CI electrode insertion has been the contemporary standard of care for optimizing hearing preservation after surgery (Lenarz et al., 2020; Bautista-Salinas et al., 2022). Thus far, “soft surgical” techniques have been employed through slow manual insertions and use of flexible electrode arrays. One compelling strategy to enhance “soft surgical” technique is the use of robotics to assist electrode array insertion, which minimizes intracochlear forces, pressure transients, and trauma through slower and steadier insertion (Banakis Hartl et al., 2019; Kaufmann et al., 2020). While limiting the surgical trauma as much as possible is necessary, many also utilize the peri-surgical local application of anti-inflammatory drugs to further inhibit the inflammatory response related to surgery. While this one-time application may inhibit the immune response to surgical trauma, sustained delivery of anti-inflammatory molecules after CI is also being explored via the use of osmotic pumps (Scheper et al., 2017; Tarabichi et al., 2021). Separately an increasingly promising approach for sustained delivery involves controlling drug release over time through elution from the implant surface (Liebau et al., 2019). In this approach, clinical trials are currently studying the inflammatory response to CI with dexamethasone (DEX) eluting from the implant surface (Chen et al., 2021).

While it is evident that the immune response to surgical trauma to CI could be temporarily blocked with soft surgery and tailored DEX treatment, it remains to be seen whether controlled DEX release over time can effectively inhibit inflammation for a meaningful duration to benefit long -term device function (Scheper et al., 2017; Liebau et al., 2019). Specifically, it is unclear whether DEX just blocks the inflammation resulting from surgical trauma, and if the immune system will eventually respond to the implanted materials and electrical stimulation, leading to the formation of a fibrotic capsule around the implant. Therefore, there are ongoing efforts to engineer approaches to limit the immune response to electrode materials (Leigh et al., 2019; Jensen et al., 2022). To understand these approaches, it is essential to provide a brief overview of the foreign body response. The foreign body response is an inevitable process that occurs in response to any material implanted *in vivo*, involving several steps, briefly: (1) non-specific attachment of serum proteins to the implant surface, (2) acute inflammation led by neutrophils that adhere to the biomaterial surface, (3) recruitment of monocytes attempting to engulf the implant by forming a layer around it, (4) formation of giant cells when macrophages cannot engulf the implant, which orchestrate fibrosis, and (5) recruitment of fibroblasts which deposit of a dense collagen matrix around the implant (Carnicer-Lombarte et al., 2021). In theory, blocking the first steps (i.e., adsorption of protein and cells) should be highly effective at blunting this response. One example is coating the implant materials with an ultra-low fouling zwitterionic thin-film hydrogel to prevent protein or cell attachment to the implant surface, thus blocking this cascade (Chen et al., 2023; Horne et al., 2023).

The third component that may contribute to intracochlear damage is electrical stimulation. For the most part, under current clinical paradigms, the levels of electrical stimulation needed to stimulate SGNs are felt to be relatively innocuous, and may even help promote SGN survival (Leake et al., 1999, 2007; Wise et al., 2011). However higher levels of electrical stimulation have been associated with loss of residual acoustic function (Kopelovich et al., 2015), perhaps by damaging the IHC:SGN synapse and peripheral processes (Kopelovich et al., 2015; Li et al., 2020), akin to damage induced by high levels of noise exposure (Furman et al., 2013; Kujawa and Liberman, 2015). Further, in guinea pigs, high levels of stimulation, well beyond those used clinically, do not result in SGN loss yet lead to increased platinum dissolution which may exacerbate the chronic inflammatory response (Landry et al., 2013; Shepherd et al., 2021). Thus, mitigating inflammation due to surgical trauma and/or the foreign body response should enable a lower level of electrical current for the implant to function (Briggs et al., 2020). In addition to reducing any potential inflammation from electrical stimulation, this could extend battery life and improve the signal-to-noise ratio of the device.

In conclusion, limiting the immune response is an essential aspect of improving CI and other electrical devices aimed at restoring hearing. To achieve this goal, next generation CI will need to (1) mitigate the immune response to surgical trauma, (2) involve ultra-low fouling materials, and (3) enable lower levels of electrical stimulation to function. If successful, these approaches could increase the number of usable channels in CI electrodes and create an environment around the implant more conducive to regenerative strategies (Carnicer-Lombarte et al., 2021). Further, diminishing the dense fibrotic capsule that typically surrounds the implant is likely necessary to enable optimal application of engineered guidance cues and neurite regeneration strategies.

### 3.3 Related regenerative medicine approaches

Genetic causes of hearing degeneration and deafness are a significant cause for patients to need CI. In theory, gene therapy could prevent further hearing loss or even restore function for genetic causes of deafness, although the application and success of these genetic approaches remain uncertain. One significant challenge lies in delivering genetic material to the inner ear, as this therapy must overcome transport barriers while specifically targeting the delicate cells of interest, such as SGNs or hair cells (Minoda et al., 2015; Ren et al., 2019). Achieving sufficient delivery to these cells of interest may prove challenging, inspired by this challenge, CI surgery offers an excellent opportunity to deliver genetic material to the inner ear and overcome the challenge of the blood-perilymph barrier. Furthermore, the CI electrode could be used to electroporate the genes of interest (Pinyon et al., 2014), thus increasing the transfection/transduction efficiency. Gene therapy in the context of CI could serve a variety of functions, including, improving function of auditory neurons, correcting a genetic mutation, or stimulating regeneration of auditory neurons from their niche (Shearer et al., 2017; Tropitzsch et al., 2023).

Related to this is use of stem cells or growth factors to regenerate the cells of interest, typically hair cells or SGNs. To achieve this, stem cells would be delivered to the cochlea, differentiated into the desired cell type, and integrated into the respective anatomical and functional niche (Roccio et al., 2020). A major challenge that hinders the use of stem cells in hearing regeneration is the complexity of the niches that need to be filled. While it may be challenging in the near future to achieve complete regeneration of the cells of interest into their proper niches, even modest integration that provides partial function could lead to significant improvements in native hearing or CI performance (Diensthuber and Stöver, 2018). Therefore, there is merit in efforts to combine stem cell-based approaches with CI. For example, as mentioned earlier, stem cells were discussed as a potential tool in combination with CI to deliver factors or act as a bridge between the electrode array and the neurons (Nella et al., 2022).

## 4 Conclusion

To conclude, CIs are a remarkably successful device, however, further challenges prevent its ability to fully restore complex auditory perception. There are many strategies to improve CI that may be achievable in both the near and the long term. Firstly, limiting the insertional trauma and the inflammatory/immune response seems to hold great potential for improving device performance in the near future (Carnicer-Lombarte et al., 2021; Rahman et al., 2022). In the long term, the field needs to pursue approaches to develop CI capable of more closely approximating natural hearing and transitioning CIs from a far-field to near-field device. Limiting the immune response to the electrode represents a significant improvement, but alone it will not enable CI to provide near native auditory sensation. Engineering the peripheral process of SGNs to be in close proximity to the CI electrodes would theoretically dramatically enhance the number of discrete, perceptible channels. It remains to be seen which techniques (e.g., surface patterning, small molecule release, hydrogel coating, optogenetics, genetic engineering, or stem cell engineering), alone or in combination, will best achieve this challenging ambition. Beyond hearing rehabilitation, CI represent an excellent device to study the general engineering principles to improve the neural-electrode interface for the rapidly emerging field of neural stimulation/modulation (Nassiri et al., 2022).

## Author contributions

JV: Writing – original draft, Writing – review & editing. AC: Writing – review & editing. MH: Writing – review & editing.
